# Testing Strategies for Detection of Xenotropic Murine Leukemia Virus-Related Virus Infection

**DOI:** 10.1155/2011/281425

**Published:** 2011-07-28

**Authors:** Shixing Tang, Indira K. Hewlett

**Affiliations:** Laboratory of Molecular Virology, CBER, Food and Drug Administration, Bethesda, MD 20892, USA

## Abstract

Xenotropic murine leukemia virus-related virus (XMRV) is a newly identified gamma retrovirus and may be associated with prostate cancer- (PC) and chronic fatigue syndrome (CFS). Since its identification in 2006 and detection of polytropic murine lenkemia virus (MLV)-like sequences in CFS patients in 2010, several test methods including nucleic acid testing methods and serological assays have been developed for detection of XMRV and/or MLV-like sequences. However, these research assays have not yet been validated and evaluated due to the lack of well-characterized reference materials. Mouse DNA contamination should be carefully checked when testing human specimens in order to avoid false-positive detection of XMRV or MLV-like sequences.

## 1. Introduction

When XMRV was first identified in PC patients in 2006 [1], it did not get much public attention until a science paper published in 2009 reported its detection in a majority (67%) of CFS patients and about 4% of healthy Americans [2]. In this report, XMRV was shown to be infectious and could be isolated from peripheral mononuclear cells (PBMCs) and plasma, indicating that it is the first gamma retrovirus that infects humans and may be associated with human diseases [1, 2]. Furthermore, if confirmed, it was thought that millions of persons worldwide may harbor the new virus and thus pose a serious concern to public health and the safety of blood transfusion and organ transplantation. These findings greatly stimulated the interest of scientists in academia and government agencies to address both public health and scientific concerns about the newly identified retrovirus and its possible association with human diseases. However, the studies that followed the original publications have yielded conflicting findings and generated more controversy than consensus about XMRV detection and its potential disease association (see reviews [3–11]). In 2010, Lo et al. reported the detection of polytropic MLV-like sequences in 87% of CFS patients [12]. MLV-like sequences are different from, but very similar to, XMRV [12]. The detection of polytropic MLV in CFS patients suggested that XMRV may be only one of an apparent cluster of MLV-like viruses identified in patient specimens. In this paper, we describe and summarize the various testing methods and assays that have been employed for detection of XMRV and/or MLV-like virus infection in the studies that have been published until the present time.

## 2. XMRV Testing Methods

A variety of test methods have been employed to detect XMRV in cell culture studies and clinical specimens. Polymerase chain reaction (PCR) assay (nested and real-time PCR), transcription-mediated amplification assay (TMA), and fluorescence *in situ* hybridization (FISH) have been used for direct detection of viral sequences. Several serologic assays for detection of circulating antibodies against XMRV have been reported, including flow cytometry (FACS), Western blot (WB), chemiluminescence-based immunoassays and enzyme linked immunosorbent assay (ELISA), and so forth. Immunohistochemical staining (IHC) has been used for direct detection of viral proteins, while cell culture assays were used for isolation and detection of infectious virus.

### 2.1. Nucleic Acid Testing (NAT)

Viral nucleic acid detection by reverse transcription- (RT-) based PCR or DNA PCR methods has been widely used for detection of XMRV and/or MLV-like sequences, but the results have generated controversial findings partly because of the differences in detection sensitivity and specificity [11, 13, 14]. Several issues need to be considered when using PCR to detect XMRV and/or MLV-like sequences. Mikovits et al. emphasized that the PCR template is critical for successful amplification and relatively high-detection rate of XMRV [15, 16]. They found that by using DNA extracted from inactivated PBMCs as template, only 7% and 21% of CFS patients were positive in single-round and nested PCR, respectively. By using viral RNA template from activated PBMC and cocultured cells and RT PCR, the detection rate was 72% and 89%, respectively [15, 16]. Using activated PBMC can significantly improve detection sensitivity because MLVs and other closely related retroviruses rely on mitosis to access the host cell chromosomes [17] and the copies of XMRV in activated cells are increased. Their results suggested that it may be more sensitive to detect viral RNA rather than proviral DNA, which was done in their original science paper [2]. The amount of DNA in the template is also critical. Danielson et al. found that XMRV was detected in 3.2% of the patients when 100–140 ng of prostate tissue DNA were used, compared with the positivity of 22.2% when 650 ng DNA were used [18]. However, Lo et al. reported that 87% of CFS patients were positive for MLV-like viral sequences when less than 50 ng of DNA per reaction and similar primers from XMRV were used [12]. The selection of PCR primers is another crucial factor that may affect detection of XMRV-specific sequences. The PCR primers previously claimed to be specific for XMRV have been found to be able to readily amplify MLV sequences from a variety of mice and some human cell lines [19–22], including polytropic MLV-like sequences identified by Lo et al. [12]. The typical 24 nucleotide deletion within the *gag* leader region of the XMRV genome is also found in some MLV sequences, endogenous retroviruses and in several mouse strains [19, 22]. In addition, Chow and Ikeda did not detect the 24-nt deletion in the *gag* leader region in their studies [14]. These results indicate that it may be difficult to design PCR primers that are exclusively specific for XMRV due to the high-sequence homology between XMRV and MLVs and even endogenous retroviral sequences. However, Schlaberg et al. reported a quantitative PCR for specific detection of XMRV by using primers from XMRV integrase gene, which are 100% conserved in 3 published XMRV sequences (VP35, VP42, and VP62) and share only 80–85% sequence identity with the most similar murine retroviruses [23]. They could consistently detect 50 copies of the XMRV proviral clone and 5 copies at 50% of hit rate [23]. Van Kuppeveld et al. also observed that both the real time PCR assay and the nested PCR assay could detect at least 10 copies of XMRV, indicating similar sensitivity [24]. Some studies found that XMRV could only be amplified by *gag* primers but not *env* or *pol* primers, or vice versa; or that detection sensitivity relies on primer sequences and locations. Danielson et al. reported that XMRV *gag *primers were at least 10-fold less sensitive than *env *primers; and *pol *primers tended to amplify a competing region from the human genome. They could not detect XMRV in patient tissue samples by nested RT-PCR with primers specific for *gag *and *pol *genes, regardless of whether 100 or 650 ng of DNA was used as template [18]. Stoye et al. reported that the XMRV *env* primers yielded the most positive results [14]. However, Lo et al. found *gag* primers were more sensitive than *env* primers when detecting MLV-like sequences [12]. Oakes et al. found that 53% (19/36) of the healthy volunteers and 1.8% (2/112) of the CFS patients yielded PCR products when using XMRV *gag* primers, but no positive amplification was observed when using qPCR with *pol* primers [20]. Switzer et al. [36] tested 162 PC patients and found that PCR products were obtained for *gag, pol, *and* env* from one patient, from *pol *and* env* from a second, and *pol* alone from a third case. Furthermore, PCR was not successful in all replicates on individual samples, indicating that multiple primer sets and repeats may be necessary for accurate detection of XMRV, possibly due to the very low viral titers in clinical samples [14]. The real reasons are not clear due to lack of consensus results. Although XMRV sequences appear to be highly conserved, variations have been consistently mentioned and may account for some of the negative results [5, 15, 25].

A major concern in regard to XMRV detection by PCR is false-positive results caused by contamination. Recently, four publications that appeared in the journal Retrovirology identified three potential sources of contamination in PCR-based studies of XMRV. Robinson and Oakes et al. reported independently that all XMRV-positive samples in their analysis were also positive for mouse DNA when assayed using a mouse mitochondrial DNA PCR or intracisternal A particle (IAP) assay [19, 20]. Hue et al. confirmed that 2.2% (5/411) of human cell lines screened were positive for MLVs [22]. Sato et al. found that commercial RT-PCR reagents were contaminated with MLV RNA [21]. The contamination was thought to originate from the hybridoma cell line from which the monoclonal antibody used in the polymerase reaction mixture to facilitate hot-start PCR was prepared. These results indicate that mouse DNA contamination is widespread and can confound XMRV detection in human samples. Furthermore, Hue et al. compared the published XMRV sequences with those from XMRV positive 22Rv1 cell, and found the genetic distance among 22Rv1-derived sequences exceeds that of XMRV sequences from patients, indicating that XMRV detected in patients may result from laboratory contamination rather than a true human infection [22]. Interestingly, 22Rv1 cell line was derived from a human prostate cancer xenograft (CWR22) that was serially passaged in nude mice in 1990s. Paprotka et al. recently reported that XMRV was the recombinant of two endogenous MLVs during passage of the CWR22 PC xenograft [26], suggesting that XMRV is a laboratory-derived virus and may have contaminated samples for more than a decade, but it may not infect people. These results clearly show that extraprecautions must be taken to avoid mouse DNA contamination and false-positive amplification from human cell lines that harbor xenotropic MLVs since they are closely related to XMRV. PCR methods have been used to detect mouse mitochondrial DNA [12], cox2 DNA [20], and IAP [19, 20]. IAPs are endogenous transportable elements present at the level of about 1000 copies per mouse genome [27]. Robinson et al. found the mtDNA PCR was 100-fold less sensitive than that for IAP when testing in both McCoy cell and RAW 264.7 cell DNA [19], suggesting that IAP PCR may be more suitable for finding contamination of murine sequences. 

Due to concerns about variations in nucleic acid-based methods that have been used to study XMRV, the National Heart, Lung, and Blood Institute (NHLBI) under the leadership of the Health and Human Services (HHS) sponsored an XMRV Blood Scientific Working Group (BSWG) to validate the testing assays that have been developed and used in different laboratories for CFS and blood donor testing [28]. Preliminary results of this working group indicated that most nucleic acid testing assays used in different labs were able to achieve similar levels of sensitivity and specificity based on the spiked XMRV panels [28]. However, when testing CFS samples, no consensus of results was observed. Therefore, it is still not clear if assay methodology alone could account for the large differences observed when testing clinical samples.

### 2.2. Serology

Antibody detection is considered as strong evidence of XMRV infection, in particular, in the absence of positive PCR results, or when the PCR results are not very reliable due to differences in assay sensitivity and specificity. High-throughput serologic assays that detect XMRV-specific antibodies would be of great value for determining the epidemiology of possible XMRV infection and for addressing the association of XMRV infection and human diseases if they exist at all. However, the nature and kinetics of the antibody response to XMRV infection have not been well-characterized; or it may be premature to discuss the antibody responses since XMRV infection of humans has yet to be proven.

Lombardi et al. first reported on specific immune responses to XMRV in CFS patients [2]. They used FACS to measure the antibody against XMRV by mixing patient plasma with a mouse B cell line expressing recombinant spleen focus-forming virus (SFFV) *env* protein. Because XMRV shares >90% overall nucleotide sequence identity with known MLVs, cross-reactivity between anti-MLV antibodies and XMRV proteins has been observed and 50% (9/18) of CFS patients were shown to be reactive [2]. Furthermore, anti-XMRV-positive plasma samples from CFS patients blocked the binding of anti-SFFV *env* antibody to SFFV *env* on the cell surface. They claimed that FACS was one of the most sensitive blood-based assays for detection of anti-XMRV *env* antibody in patient plasma and could detect 82% (47/57) of XMRV infection in CFS patients [15, 16]. Arnold et al. developed a single-round reporter gene based on neutralizing antibody assay and found that 27.5% (11/40) PC patients were anti-XMRV antibody positive [29]. In their system, anti-XMRV antibody was confirmed by blocking the binding and infection of XMRV-HIV pseudovirus expressing XMRV *env* protein with the reporter cell line JC53BL-13 that carries the firefly luciferase and *β*-galactosidase under the control of the HIV-1 LTR. The results were in complete concordance with both PCR and FISH for 7 patients in whom 3 assays were conducted [29]. However, Sabunciya et al. did not find differences in immunoreactivity between PC patients and controls by ELISA using XMRV *env* or *gag* antigen to capture anti-XMRV antibodies [30]. A similar neutralizing antibody assay developed by Groom et al. could not detect specific anti-XMRV antibody in 170 CFS patients [31]. These results highlight the controversy in detecting anti-XMRV antibodies due to the lack of validated assays and well-characterized reference materials. 

Onlamoon et al. infected rhesus macaques with XMRV to develop an animal model of XMRV infection and to investigate the viremia and immune response against XMRV [32]. They found that viremia was detected 4 days postinfection (PI) with a peak on day 7 for one animal and a delayed and lower acute viral replication kinetic at days 14–20 in another animal [32]. The antibody responses to XMRV were observed during the second week of infection and boosted upon reexposure, but titers decreased rapidly [32, 33], indicating a weak immune response. The predominant responses were to the envelope protein gp70 and p15E, and capsid protein p30 with higher titers to gp70 and p15E than to p30, especially in acute infection of XMRV. Antibodies to gp70, p15E, and p30 persisted to 158 days and were substantially boosted by reinfection, indicating that they may be useful serologic markers for XMRV infection [33]. Based on these findings, direct chemiluminescent immunoassays (CMIAs) on the automated ARCHITECT instrument system were developed by using recombinant proteins (p15E, gp70, and p30) for both capture and detection. The prototype assays showed 100% sensitivity by detecting all Western Blot positive serial bleeds from the XMRV-infected macaques and >99% specificity with blood donors [33]. This is the first study to investigate the nature and kinetics of the antibody response to XMRV infection in animal model, and the first immunoassay that has been evaluated by the well-characterized XMRV-positive animal bleeds. 

The US National Cancer Institute (NCI) has invested considerable effort in developing serological assays for detection of XMRV infection. They used the Meso Scale Discovery (MSD) platform and found that reactivity to XMRV recombinant proteins is statistically higher in XMRV-positive clinical samples from CFS patients than in normal donor plasma [34]. Conventional ELISA has been developed by using recombinant XMRV-proteins to capture antibodies in samples [30, 35]. In addition, several groups reported the use of the WB assay for detection of anti-XMRV antibodies [36, 37]. 

Unfortunately, all of these assays have not yet been fully validated due to lack of reference reagents for standardization of assays; therefore, it is difficult to make comparisons of results obtained in different laboratories using various-methodologies. Several groups were unable to detect XMRV-specific antibodies in PC and CFS patients as well as healthy control sera [30, 35, 36, 38]. In some studies, the weak antibody activity could not be confirmed by other assays, such as immunofluorescence assay (IFA) or was found to be nonspecific cross-reactivity [38]. Groom et al. found that only one of the 26 samples with neutralizing activity came from a CFS patient, while 14% (22/157) blood donors were positive by their neutralizing assay [31]. However, most of the serological positive samples were also able to neutralize MLV particles pseudotyped with enveloped proteins from other viruses, including vesicular stomatitis virus, indicating significant cross-reactivity in serological responses. These results highlight the danger of overestimating XMRV frequency based on serological assays.

### 2.3. Other Test Methods

Both FISH and IHC were employed to localize XMRV within human prostatic tissues, and to measure the frequency of the infected cells. Urisman et al. reported that about 0.1~1.2% of the cells were positive and most FISH-positive cells were stromal fibroblasts [1]. They also used IHC to stain PC tissues with a monoclonal antibody against SFFV that was reactive against *gag* proteins from a wide range of different ecotropic, polytropic, and xenotropic MuLV strains. However, they observed that only stromal but not epithelial cells were infected with XMRV [1]. In contrast, Schlaberg et al. stained prostate tissues with anti-XMRV sera and found viral protein expression in 23% (54/233) of cases with PC and in 4% (4/101) controls. XMRV-specific staining was predominantly observed in malignant prostatic epithelial cells [23]. However, only 6 out of 54 IHC-positive PC cases were XMRV DNA positive while none of the controls was positive for the two assays. Differences between the two methods were argued to be attributed to very low viral loads, sampling differences, and varying proportions of XMRV-infected cells [23]. Aloia et al. reported no staining of 596 prostatic adenocarcinomas and 452 benign prostate tissue specimens by IHC using anti-MLV antibodies which reproducibly reacted with XMRV proteins and stained XMRV-containing cells [39]. They were unable to stain positive samples from the Schlaberg study, raising the question of whether the findings by Schlaberg et al. were valid [39]. Barnes et al. used T-cell ELISPOT assay to test responses of PBMCs to XMRV *gag* peptides as an evidence of XMRV infection of PBMCs [40]. For *ex vivo* T-cell ELISPOT assays, microtiter plates coated with anti-interferon-*γ* immunoglobulin G were used to incubate with XMRV peptides and PBMCs from patients, followed by incubation with biotinylated interferon-*γ* antibody and streptavidin-labeled chromogen. The number of reactive cells was counted. They found no positive responses to XMRV *gag* in the 63 patients infected with HIV-1 or HCV. However, patient cells were responsive to other antigens, suggesting that the absence of XMRV *gag*-specific T cells in their study. Lee described a novel cell line, Detectors of Exogenous Retroviral Sequence Elements (DERSE) indicator cells, to rapidly assess XMRV or XMLV replication in less than 3 days [14]. The DERSE cell uses a retroviral vector containing an inverted, intron-interrupted green fluorescent protein (GFP) reporter cassette. XMRV infection permitted GFP expression, which can be easily monitored by microscopy.

## 3. Conclusion

Currently, there are no commercially available FDA approved/licensed tests for detection of XMRV or other MLV-related human retroviruses. Standards for the diagnosis of XMRV or MLV-related retrovirus infection based on laboratory test methods have not been established. The relative sensitivity and specificity of various assay methodologies and strategies (i.e., NAT, serology, and culture) have not been determined and standards for assay performance have not yet been established. The use of multiple testing methodologies may be required because of the biology of the viruses, such as transient viremia and relatively low-immune response observed in the Macaque model. In order to avoid false-positive detection, mouse DNA contamination should be carefully examined and excluded.
